# Supplementary material for: A Fluorescent Nanosensor for Silver (Ag+) and Mercury (Hg2+) Ions Using Eu (III)-Doped Carbon Dots
Source: Nanomaterials (Basel). 2022 Jan 25;12(3):385. doi: 10.3390/nano12030385 (PMC8838628; doi:10.3390/nano12030385)
Supplement: Supplementary file 1 [file nanomaterials-12-00385-s001.zip › nanomaterials-1564913-supplementary.pdf]

Electronic Supplementary Information

Article

A fluorescent nanosensor for silver (Ag^{+}) and mercury (Hg^{2+}) ions using Eu(III) doped carbon dots

Cátia Correia, José Martinho and Ermelinda Maçôas*

Centro de Química Estrutural (CQE) and Institute of Molecular Science, Instituto Superior Técnico, Universidade de Lisboa, 1049-001 Lisboa, Portugal; catia.correia@tecnico.ulisboa.pt (C.C.), jgmartinho@tecnico.ulisboa.pt (J.M.), ermelinda.macoas@tecnico.ulisboa.pt (E.M)

* Correspondence: ermelinda.macoas@tecnico.ulisboa.pt

Table S1. High resolution XPS spectra of the C1s and O1s peaks of the Cdots and Eu-Cdots with the attribution to functional groups and the corresponding atomic percentages.

	BE (eV) Cdots (Eu-Cdots)	assignment	Cdots (%)	Eu-Cdots (%)
C1s,	285.0 (285.0)	C=C/C-C	53.8	42.4
	286.8	C-OH/C=O	17.8	
	(286.6)	COH/COC		16.5
	288.5	O-C=O/N-C=O	27.7	
	(288.6)	COO^{-}…Eu^{3+}		29.5
	290.2	O-COO^{-}	0.7	
	(289.7)	O-COO^{-}….Eu^{3+}		11.6
O1s	531.5	C=O		
	(531.6)	$C\underline{OO}^{-}$…Eu^{3+}/	58.5	
		O-$C\underline{OO}^{-}$…Eu^{3+}		81.5
	532.6	COH	32.8	
	(532.8)	$\underline{O}$-COO^{-}….Eu^{3+}		14.7
	533.5 (534.0)	H_2O	8.7	3.8
N1s	398.4 (398.7)	sp^2 C-N=C	10.1	6.5
	399.9 (400.2)	sp^3 >N-C=O	87.5	67.5
	401.4 (402.0)	>N^+=C, -NH^+_3	2.4	26.0

Table S2. Decay parameters of the C-dots and Eu-cdots emission in the absence and presence of 100 μM of Ag^+ and Hg^{2+}

Decay parameters	Cdots	Eu-Cdots	Eu-Cdots + Ag^+ (100 μM)	Eu-Cdots + Hg^{2+}(100 μM)
τ_1/ns (a_1)	3.11 (0.40)	3.00 (0.26)	2.19 (0.34)	4.78 (0.21)
τ_2 /ns (a_2)	8.60 (0.20)	7.90 (0.23)	8.50 (0.14)	12.27 (0.53)
τ_3 /ns (a_3)	0.67 (0.40)	0.67 (0.52)	0.50 (0.52)	1.00 (0.26)
τ_{av}/ns	3.23	2.95	2.19	7.77
χ^2	1.0	1.0	1.1	1.1

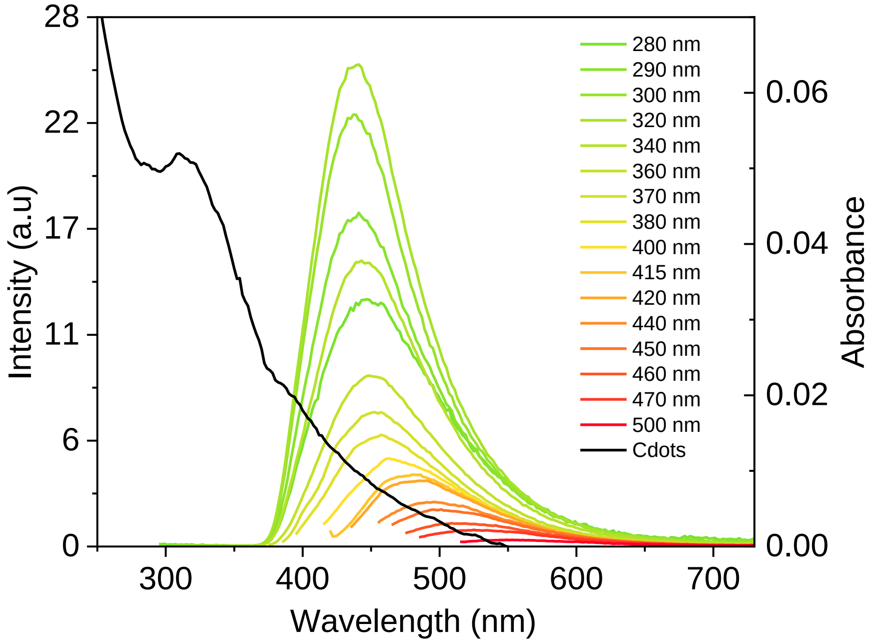

Figure S1. Absorption spectrum (black) and luminescence spectra (green to red) of undoped Cdots. Excitation at several wavelengths is shown.

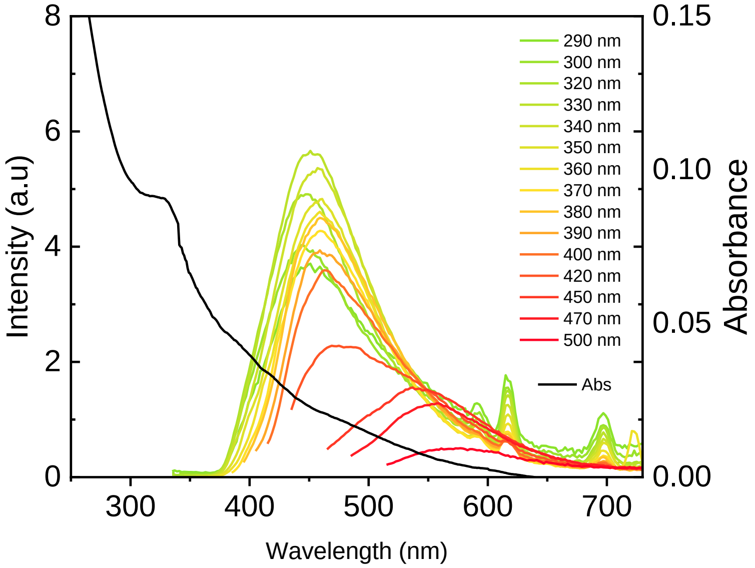

Figure S2. Absorption spectrum (black) and luminescence spectra of the Eu-Cdots upon excitation at several wavelengths (green to red).

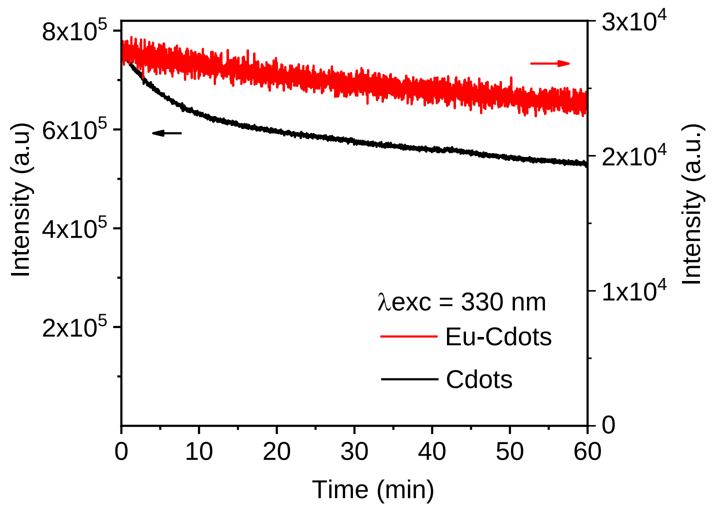

Figure S3. Photostability of Cdots and Eu-Cdots. Emission intensity of Cdots (black) and Eu-Cdots (red) under irradiation at 330 nm.

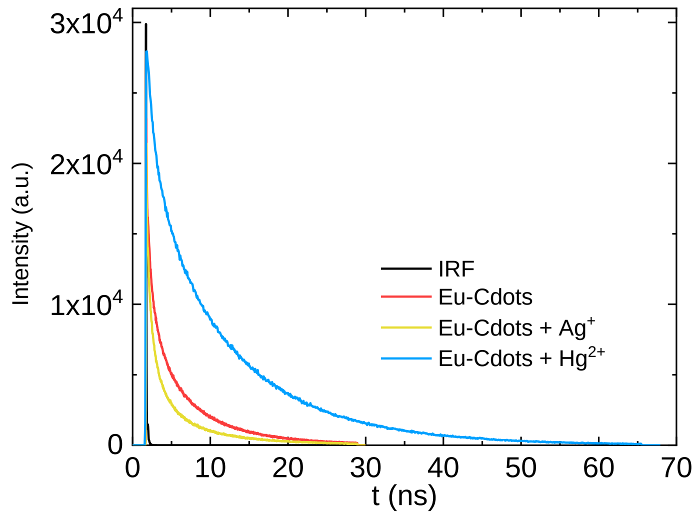

Figure S4. Emission decay curves for Eu-Cdots (red), and Eu-Cdots in the presence of 100 mM of Ag^+ (yellow) and Hg^{2+} (blue). The Instrumental response function (IRF) is shown in black.
